# Special Issue: “Biophysics, Arrhythmias and Pacing”

**DOI:** 10.3390/biology12040569

**Published:** 2023-04-08

**Authors:** Matteo Bertini

**Affiliations:** Cardiology Unit, Azienda Ospedaliero Universitaria di Ferrara, Cona, 44124 Ferrara, Italy; matteo.bertini@unife.it; Tel.: +39-5322-36269

Cardiac pacing technologies have been implemented during the last few decades, including leadless pacemakers and pacing of the conduction system, such as His bundle pacing and left bundle branch area pacing. Furthermore, cardiac pacing has improved the possibility of monitoring patients and developing new treatments as well as follow-up strategies.

The continuous monitoring of cardiac implantable electronic devices (CIEDs) enables us to detect atrial high-rate episodes (AHREs) in patients with no history of atrial fibrillation (AF) [1,2]. In a recent study, AHREs of long duration were found to be strongly linked with a new AF diagnosis, clinical AF, and AHREs >24 h at a two-year follow-up. These findings may aid in the intensification of monitoring and decision-making in patients with AHREs and specific baseline characteristics [3].

Nowadays, we have cardiac resynchronization therapy defibrillators equipped with a combination of multiple sensors, allowing heart failure (HF) patients to be monitored at home. The detection of AHREs in CIED patients using the HeartLogic index is significantly associated with algorithmic alarm, regardless of event duration [4]. Furthermore, during the COVID-19 pandemic, this tool was useful in facilitating home monitoring of HF patients. There was a significant drop in the median activity level and a higher rate of alerts, suggesting a worsening of HF [5].

Another important factor to consider is that, while the complexity of devices and patients undergoing CIED implantation has significantly increased over the last three decades, CIED infections have also increased, with a lifetime incidence of 1–3% [6]. This rate varies depending on the characteristics of the patient, the operator, and the device. Several studies are investigating potential predictors of CIED infections and CIED-related complications [7,8], which can have a significant impact on patient prognosis [9]. According to the results of the multicentric ELECTRa (European Lead Extraction ConTRolled) registry [10], despite a high intraprocedural success rate, in-hospital overall mortality was not negligible. Furthermore, systemic infections are associated with a higher incidence of in-hospital and procedure-related complications, as well as overall in-hospital mortality [11].

However, whether cardiac pacing is projected into the future, the field of electrophysiology is also changing and modernizing a lot. Many technological advances have been observed in the last two decades. Catheter ablation procedures have advanced significantly and have proven to be effective even in the context of rare genetic diseases. This Special Issue focuses on β-thalassemia and cardiolaminopathies, which are associated with a higher arrhythmic and thromboembolic risk when compared to the general population.

Because of a baseline altered hemodynamic profile due to anemia, AF in β-thalassemia is rarely tolerated. It necessitates not only disease-specific therapy (e.g., chelation therapy), but also an aggressive and early rhythm control strategy [12]. Atrial and ventricular arrhythmias and conduction disturbances are common in cardio laminopathies, even at a young age. This condition has severe features, such as a malignant natural history, end stage HF, and the need for a heart transplant [13].

With the increasing use of AF catheter ablation therapy and a broader spectrum of concomitant diseases, innovative arrhythmia management techniques that offer safer and more precise ablation therapies are required. Zerofluoroscopy AF ablation represents a modern approach to treating this arrhythmia. The development of catheters as well as sheaths visible to 3D electroanatomical mapping systems allows for a completely fluoroless approach, even during transseptal puncture. The procedure demonstrated good efficacy in an interesting case series, reducing procedural time and increasing ablative precision with the use of a high-density mapping catheter [14].

Finally, several advances have been made in the acquisition of vascular accesses, both during CIED implantation and electrophysiological procedures: an Italian survey on common practice highlighted the widespread use of ultrasound guidance for venous and arterial puncture as well as the emerging use of vascular closure devices [15].
